# Detection of Genetic Variations in Coagulopathy-Related Genes Using Ramified Rolling Circle Amplification

**DOI:** 10.1155/2014/641090

**Published:** 2014-03-02

**Authors:** James H. Smith, Miao Cui, David Y. Zhang, Thomas P. Beals, Fei Ye

**Affiliations:** ^1^Research and Development Department, Thorne Diagnostics Inc. 100 Cummings Center, Beverly, MA 01915, USA; ^2^Department of Pathology, Icahn School of Medicine at Mount Sinai, 1428 Madison Avenue, New York City, NY 10029, USA

## Abstract

We evaluated single nucleotide polymorphism (SNP) detection via a target-capture, C-probe ligation, and RAM assay in a single-blind comparison to clinical samples that had been tested with FDA-cleared tests for up to 4 different vascular disease-related SNPs. In the RAM assay circulizable linear probes (C- or padlock probes) were annealed directly to genomic DNA, processed on a largely automated platform, and ligated C-probes were amplified by real-time RAM. After allele determinations were made with the experimental system, the sample genotypes were unblinded and the experimentally determined genotypes were found to be completely consistent with the FDA-cleared test results. The methods and results presented here show that a combination of C-probes, automated sample processing, and isothermal RAM provides a robust, and specific, nucleic acid detection platform that is compatible with automated DNA sample preparation and the throughput requirements of the clinical laboratory.

## 1. Introduction

The ability to distinguish single nucleotide differences between nucleic acid targets, for example, single nucleotide polymorphisms (SNPs), is a stringent challenge for a nucleic acid target detection assay. An assay platform with high specificity in SNP target detection, suggests utility for other applications where targets exhibit larger differences, such as deletions, insertions or substitutions of more than one nucleotide. In an earlier study [1], using commercially obtained control genomic DNAs, we demonstrated that a substantially automated RAM assay specifically detects all three genotypes of a SNP in the FV gene (FV 1691 G-A) [2].

To further evaluate this RAM assay platform we tested additional SNP targets in clinical samples. We chose a clinically relevant panel of 4 SNP targets (FV 1691 G-A [2], PT 20210 G-A [3], MTHFR 677 C-T [4] and MTHFR 1298 A-C [5]) that are associated with coagulopathic disease and that are routinely tested for in our laboratory using commercial FDA-cleared kits.

We tested the aforementioned SNP targets to further assess the performance of the RAM assay on additional targets, with dissimilar nucleotide substitutions, using previously characterized patient samples. Genotype correlation with vascular disease was an early application of genetic diagnosis [6, 7] and hundreds of vascular disease candidate genes have now been identified [8].

The Factor V (FV) gene mutation, FV 1691 G-A, results in an altered gene product that is more resistant to cleavage and is associated with venous disease [2]. In the clotting cascade, activated FV binds to activated Factor X to convert Factor II (prothrombin, (PT)) to an active protease that cleaves fibrinogen to fibrin (reviewed in [9]). The PT 20210 G-A mutation in the PT-encoding gene that results in elevated plasma prothrombin levels [3] is also associated with vascular disease [7].

Mutations in the above-mentioned genes encoding coagulation-related gene products are largely confined to coagulation-related phenotypes. By contrast, isoforms of the methylenetetrahydrofolate reductase (MTHFR) enzyme have been implicated in diverse phenotypes. The MTHFR 677 C-T mutation [4] that results in a thermolabile enzyme and the MTHFR 1298 A-C mutation [5] have been associated with phenotypes ranging from coronary artery disease [6] to miscarriage [10] to diabetes and obesity [11, 12].

DNA amplification from single-stranded DNA (ssDNA) molecules (referred to here as C-probes) that have been circularized on targets has been used in a variety of assay formats. C-probes are linear molecules with target-specific termini separated by an internal spacer sequence or core and have been described by us [1, 13] and others [14–17]. Following target-dependent ligation the resulting ssDNA circles can be amplified via ramified DNA amplification using a pair of primers [15, 17], a single primer [18], or by polymerase chain reaction (PCR; [15]).

Here we evaluate the C-probe ligation and RAM amplification assay as previously described [1] for highly specific nucleic acid detection by testing clinical samples for up to 4 SNP targets. Employing capture-probe technology on an automated platform eliminated RAM reaction noise when ssDNA circles were detected by SYBR-Green fluorescence in real-time amplification reactions. In a single-blind comparison, we tested patient genomic DNA samples, from whole blood, for the aforementioned coagulopathy-related polymorphisms. After RAM testing was complete, sample genotypes were scored as wild type (Wt) or mutant (Mt) homozygotes, or as heterozygotes. Sample genotypes were then unblinded and compared to RAM results.

## 2. Materials and Methods

### 2.1. Methodology Overview

**Figure 1:**
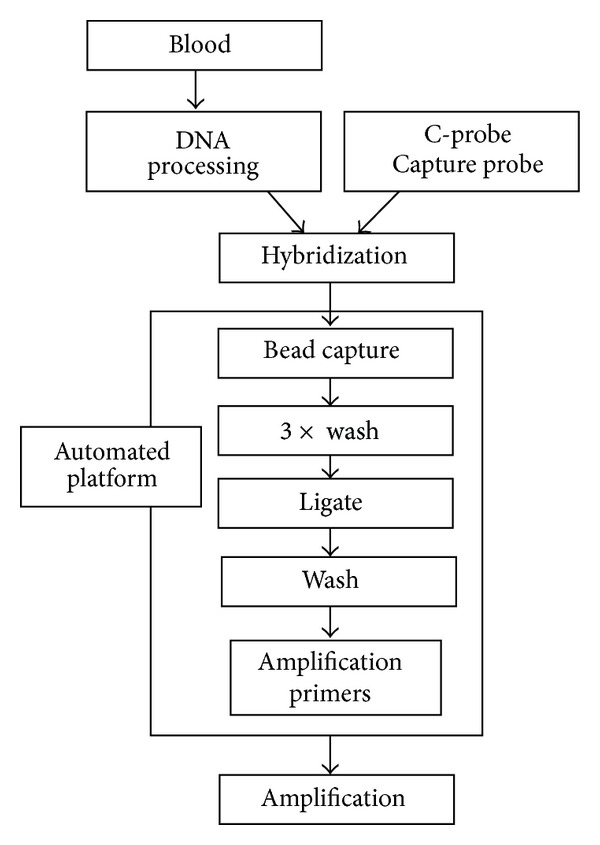
Process diagram for RAM assays. The figure shows conceptually the groups of operations performed as described in the text. “Hybridization” refers to DNA hybridization. “Automated platform” refers to the Kingfisher 96 instrument described in the text.

The workflow for the experimental assay was designed to be compatible with the automated whole-blood sample processing protocols employed prior to analysis with commercial assays.  Figure 1 shows an overview of the RAM-assay-based process described here; a more detailed description was provided earlier [1]. DNA samples were fragmented and then incubated with SNP-specific C-probes and biotin-tagged capture probes under DNA hybridization conditions as described earlier [1]. After hybridization, binding of the capture probe/target/C-probe ternary complex to streptavidin-coupled magnetic beads, bead-washing, C-probe ligation, and sample suspension in RAM-assay-ready form followed as automated steps. Real-time signals were recorded for each RAM reaction via SYBR-Green fluorescence [13] monitoring.

### 2.2. Reagents

Hybridization, wash, and elution buffers and ligation, amplification, and bead mixtures were described earlier [1]. The beads were SeraMag Streptavidin Particles (part No. 3015105010150, Thermo Scientific, Indianapolis, IN) with a nominal biotin-binding capacity of 4559 pmol biotin/mg.

### 2.3. Nucleic Acids Detection Reagents Design and Quality Control

**Figure 2:**
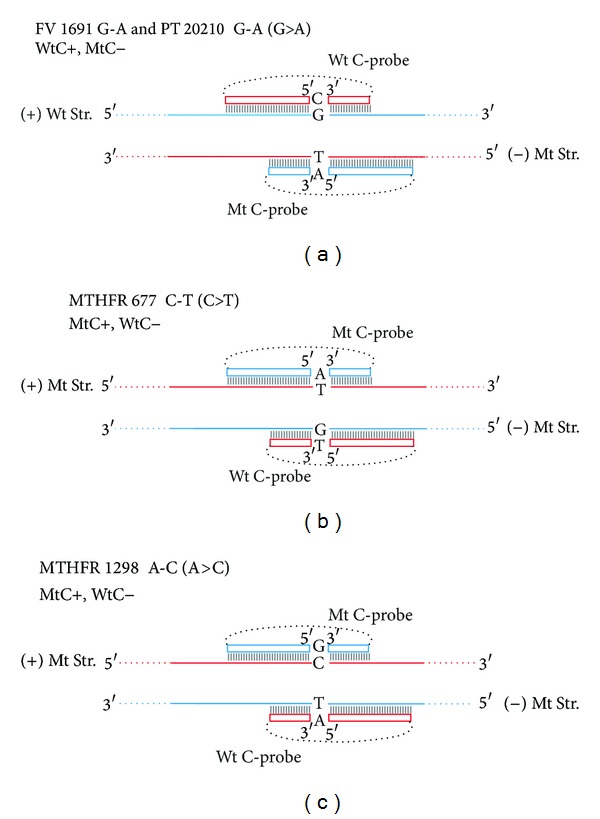
The alignment of C-probe target specific termini that detect SNP targets via the RAM reaction. The figure shows Wt target strands (“Wt Str.”) and Mt C-probe target-specific terminal sequence regions in blue and the Mt target strands (“Mt Str.”) and Wt C-probe terminal target specific regions in red. Wt and Mt C-probe termini are shown as if bound to their respective targets, and joined by a target-non-homologous linker represented by a dotted line. The SNP base is shown in bold-face in the target strands and on the 3′ terminal base of each C-probe. Strand specific capture probes (not show) bind proximally downstream (3′) of the C-probe binding domain. (a) shows the FV and PT target/probe arrangement, WtC+, MtC−, and (b and c) the MtC+, WtC− format used for the detection of both MTHFR SNPs, MTHFR 677 C-T and MTHFR 1298 A-C respectively.

All C-probes, capture probes and RAM primers were synthesized by Gene Link Inc. (Hawthorne, NY), and both C-probes and capture probes were gel-purified by the vendor. All sequences are shown in Table 1. Capture probes were modified with a 5′ biotin moiety (Table 1 “Bio-”) for streptavidin capture and with a 3′ spacer C3 moiety (Table 1, “-Spacer C3”) to block 3′ extension by the DNA polymerase.  Figure 2(a) depicts conceptually the alignment of the target-specific termini of a pair of C-probes that detect SNPs on the Wt and Mt DNA target strands. 2 strand-specific biotin-labeled capture probes (not shown ([1], Figure 1)) bind to each strand 3′ proximal (downstream) to the C-probe binding domain. The biotin moiety on the capture probe mediates binding of the ternary complex (target/C-probe/capture probe) to streptavidin-coated beads.

C-probes with allele-specific termini were designed as described earlier [1]. As described below, there are several options for the design of probe-target recognition and the two employed in this study are illustrated in Figure 2. C-probe, DNA-target combination designations indicate the target strand (plus or minus strand, or top or bottom strand, etc.) to which the C-probe binds, and a target-allele indicator (here, Wt or Mt). We indicate a combination by writing the target form, followed by the letter “C” to indicate a C-probe, followed by a target-strand indicator. For example, WtC+ indicates a C-probe that binds the target plus-strand to detect a wild-type genotype. Multiple pairings are comma-separated; thus, the combination of WtC+, MtC− adds to the above a mutant-detecting C-probe that is complementary to the “minus” target strand (Figure 2(a)) and a second combination, MtC+, WtC−, is illustrated in Figures 2(b) and 2(c). For another example, an alternate version of the assays described here could utilize a single capture-probe and two C-probes that are specific for alternate forms of one target strand: WtC+, MtC+ (configuration not shown). The potential advantage of these options is described in the Discussion Section.

The C-probe design-phase included compatible primer specification; initial primers were selected using Primer3 [19]. Selected C-probes and primers were synthesized and primer pairs were tested in real-time RAM assays with preformed circularized C-probes as templates (data not shown). C-probe concentrations in hybridization reactions (Table 2) reflect adjustments for specific C-probe performance and for synthesis-batch-specific C-probe characteristics as described earlier [1]. Primer pair concentrations in RAM reactions (Table 2) reflect synthesis batch-specific optimization and reaction rate characteristics, as described previously [1].

Biotin-linked capture probes provide an additional measure of specificity by annealing to a defined sequence flanking the SNP locus of interest. The target-specific capture probe sequences (Table 1) are designed to bind to target strands nonpreferentially with respect to the SNP locus genotype.

### 2.4. Clinical Samples, DNA Preparation, and Control DNA

Blood samples were obtained from patients presenting for thrombosis or related conditions at the Molecular Pathology Laboratory, Icahn School of Medicine at Mount Sinai. The governing Institutional Review Board determined that this work was not subject to regulation as determined by Department of Health and Human Services and Food and Drug Administration policies. Patients' DNA was isolated from 50 uL (PT and FV) or 200 uL (MTHFR) of blood using a Magnapure LC Instrument (Roche Applied Science, Indianapolis, IN) and QIAamp DNA blood mini kit (QIAGEN, Valencia, CA), respectively. DNA was eluted in 100 uL elution buffer (Magnapure) or 200 uL elution buffer (QIAGEN). Typical DNA concentrations were from 10 to 20 ug/mL [20].

**Figure 3:**
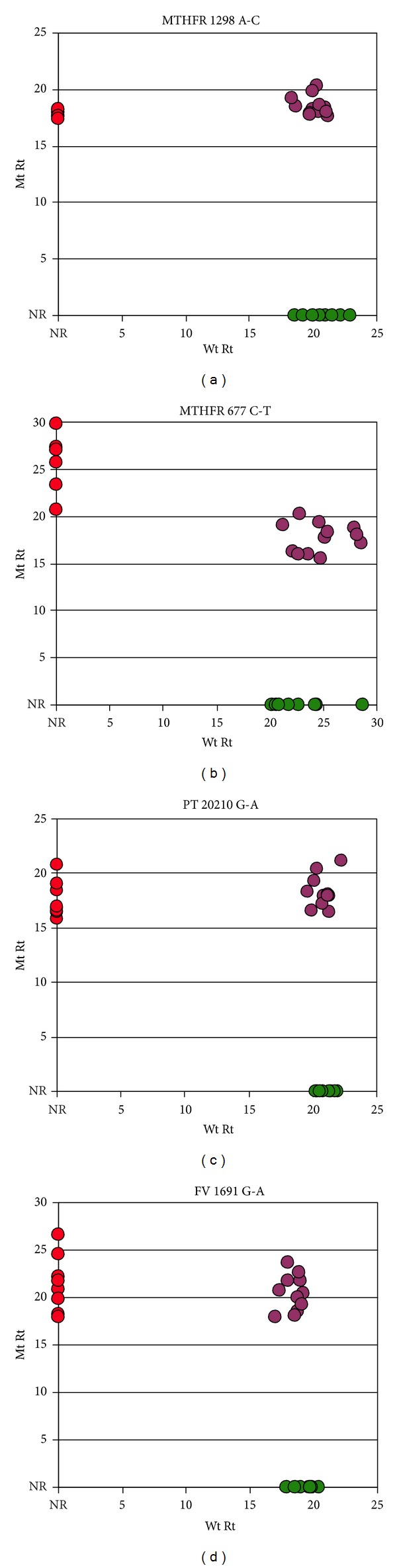
Representative plots of RAM assays performed on control DNA samples. The data points represent RAM signals (Rts) from C-probes ligated to ~6 × 10^3^ genome equivalents. Green data points represent Wt samples (wild type response times, “Wt Rt”); red data points represent homozygous Mt samples samples (mutant response times, “Mt Rt”); purple data points represent heterozygotes. “NR” on the axis scales represents “No Response”. Each homozygous sample response time is plotted on its labeled axis and at the no-response level on the alternate axis; for example, wild type (Wt) data points are plotted at on the “Wt” axis and at “NR” on the “Mt” axis. “Rt”, response time. MTHFR 677 C-T “Mt” homozygous samples done in a separate assay have a higher mean response time than the same C-probes applied to the heterozygous samples. Separate measurements for each allele from heterozygous samples are plotted within the panels as example pairings of allele measurements.

Controls were obtained from the Coriell Institute for Medical Research (Camden, NJ). Coriell catalog ID numbers and the corresponding C-probes used for SNP detection are listed in Table 3. For control assays (Figure 3), 4 × 10^4^ genome-equivalents of Coriell DNAs were used per hybridization.

We expect from the central limit theorem that C-probe circle numbers in postligation control samples are normally distributed; since response time is a logarithmic function of circle number [13] we fit control response times to a log-normal distribution. Statistical evaluation was done in the R statistical computing environment [21]. Control data sets were initially evaluated for log-normality by a Shapiro-Wilks test; data sets with *P* values less than 0.05 were tested for outliers by Grubbs' method (http://www.CRAN.R-project.org/package=outliers [22]). Grubbs test data points with *P* values less than 0.05 were identified as outliers and removed, and then the data was retested.

The genotypes of the clinical samples had been determined using the following commercial assay reagent kits. FV and PT genotypes were determined using Factor V Leiden (FVL) (Leiden FV 1691 G-A) and Factor II (Prothrombin PT 20210 G-A) kits (Roche Diagnostics, Indianapolis, IN) respectively. These tests are performed by real-time PCR assays followed by melting curve analysis with fluorescence resonance energy transfer (FRET) probes targeted at the FVL or PT mutation sequences. MTHFR 677 C-T and MTHFR 1298 A-C mutation testing were performed using Hologic's Invader Assay from Third Wave Technologies (Madison, WI).

### 2.5. Sample Processing for RAM Assays

66 uL DNA, eluted as described above, was digested in 300 uL of 1X New England Biolabs (NEB, Ipswich, MA) restriction enzyme buffer 4 containing 30 units/mL of BsaI, BspHI, FokI and HaeIII (NEB) at 37°C for 1 hour, leaving targets of interest on DNA fragments ranging from 149 to 344 nucleotides. Samples were then denatured at 95°C for 10 minutes. 34 uL aliquots of the digest per individual assay were combined, with mixing, with 23 uL of 2.5x hybridization buffer [1] containing C-probes and capture-probes, at concentrations specified in Table 2, in the wells of a 96-well microtiter plate. The plate was sealed and held for DNA hybridization at 52°C for one hour.

### 2.6. Magnetic Beads

Preparation of magnetic beads derivatized with streptavidin was performed according to Smith and Beals, 2013 [1]. 50 µL of beads, resuspended to 0.025% solids in 1x hybridization buffer, were added per well of a bead source plate. The final bead concentration at the binding step was 0.022%.

### 2.7. Elution Plate

Elution plates were prepared by adding 50 µL of low salt elution buffer containing C-probe-specific primers to each well. The primer concentrations for the elution buffers are shown in Table 2.

### 2.8. Automated Sample-Processing

Post-hybridization steps (binding, washing, ligation, re-washing and elution) were carried out on an automated sample-processing platform (Kingfisher 96, Thermo Fisher Scientific Inc., Newington, NH) as described earlier [1]. In the final step on the King-Fisher the ligated circles were separated from the beads in low ionic strength elution buffer containing RAM primers; the magnetic beads were subsequently discarded leaving circularized C-probes in the eluate.

### 2.9. RAM Amplification

10 uL from the eluate wells was combined with 10 uL RAM reaction mix [1] containing SYBR-Green dye (Molecular Probes, Eugene, OR). In this series of experiments approximately one fifth of the eluate was tested first, allowing duplicate amplifications to be performed on each sample to allow for systems diagnostics. Isothermal RAM reactions were performed at 63°C for 90 minutes in an iCycler (Bio-Rad, Hercules, CA) real-time fluorescence reader. Since the RAM reaction is not a cyclic process, we interpreted the cycle threshold (Ct), as reported by the default settings of the iCycler iQ version 3.1 software, as a response time (Rt; [13, 23]).

### 2.10. RAM Response-Time Postassay Interpretation

Response time quality control seeks to assess whether response times are due to variance in sample DNA content and to reject rare responses due to C-probe mismatch ligation of a fraction of C-probes [24]. The assay performance of each DNA preparation is inferred and updated from multiple allele assay results from each DNA sample. For SNP, assays subsequent measurements, in the absence of variation in DNA sample concentration, are expected to be within a two-fold range [1] of the first measurement, because the copy number of heterozygous samples should be half the copy number of homozygous samples.

**Figure 4:**
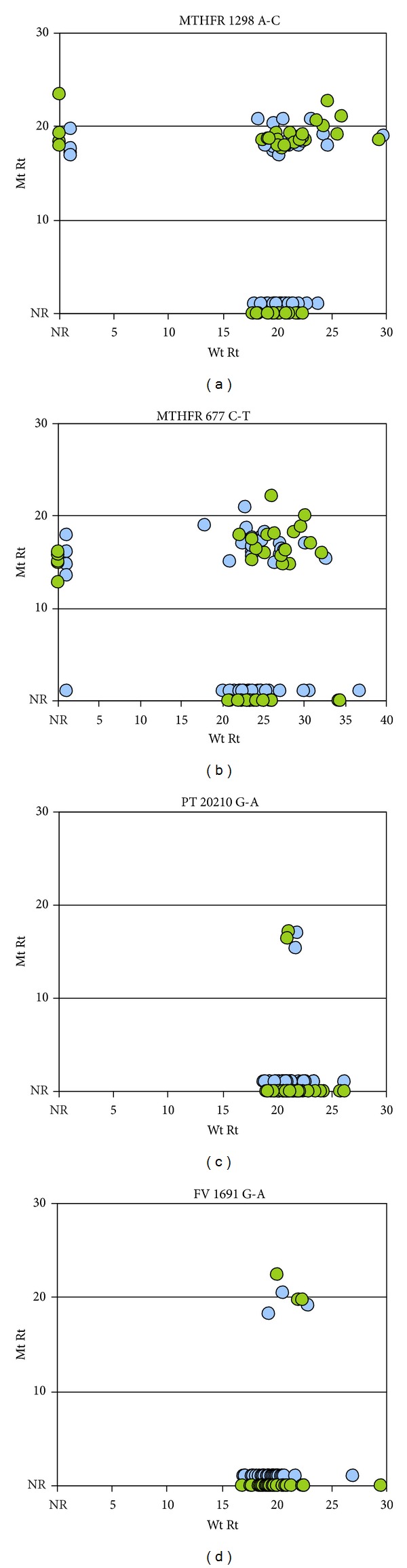
Representative plots of RAM assay results performed on clinical samples. Data points (Rts) from homozygous and heterozygous samples are represented by position as in Figure 3 (homozygous results plotted on the axes and heterozygous results appear off axes), but color distinguishes replicate RAM reactions. No homozygous Mt PT or FV alleles were detected.

Late response times that are potential mismatch ligation Rt values were evaluated based on inferred template copy number. Control reactions established a log-linear relation (e.g., [25], Figure 4(b)) between input C-probe circle number and response time, by testing preformed C-probe circle dilutions in RAM assays. The full assay-system's response (data not shown) to several levels of known-genotype genomic DNAs (listed in Table 3) established baselines for expected Rt ranges. Control reactions and Rt versus template copy number standards were constructed for each C-probe, primer-pair combination.

Response times for each C-probe annealing and each sample were scored for acceptance in the following sequence. For the assays described here, where all samples were tested for multiple loci, the first data point was accepted if it was within the expected response-time range, based on control sample data. A response-time within the expected range for any sample and either allele at any locus is taken as initial sample validation, indicating adequate initial DNA yield. Samples were scored as heterozygotes if both the Wt and Mt allele assays yielded acceptable signals. Null results for either Wt or Mt assays and an acceptable signal for the alternate allele indicated a homozygous genotype.

## 3. Results

Aliquots of sample DNA prepared as described were carried through the hybridization and automated assay process steps. Figure 3 shows the results of replicate RAM amplifications carried out on control DNAs. The response times for the Wt and Mt control samples are plotted on horizontal and vertical axes, respectively. Assays, in which genomic DNA that is homozygous at a given locus is hybridized with C-probes that are specific for an alternate allele at that locus, generally do not produce low-level noise in assays, instead usually resulting in no amplification signal. To indicate a lack of signal, the zero level of the plots in Figures 3 and 4 is labeled “NR” indicating no response.

Signals from Wt C-probes detecting Wt homozygous loci are plotted (Figure 3) at the no-response level on the “Mt” axis and at their observed response time on the “Wt” axis. Signals from assays in which Mt-specific C-probes detect homozygous Mt loci are plotted correspondingly on the “Mt” axis. In this study, C-probes for Wt and Mt loci were annealed and amplified separately. Signals from genomic DNAs that are heterozygous for the tested locus are plotted in an off-axis cluster.

We performed the RAM assay after C-probe ligation on 44 patient samples for which one or more SNP assay results had been determined using commercial kits. The RAM assays were done in single-blind fashion in the sense that the full assay comprising C-probe ligation, RAM reactions, and interpretation was performed and scored without knowledge of the commercial assay results.  Figure 4 shows graphic representations of the distribution of response-time signals for Wt response-times (Wt Rt) and Mt response-times (Mt Rt) of assay results for patient samples. To assess the consistency of repeated measures, replicate RAM assays were performed on each C-probe hybridization (shown distinguished by color in Figure 4). The number of late responding wells, out of all amplifications, was less than one percent; those readings were flagged as unrepresentative by the quality-control algorithm.

**Figure 5:**
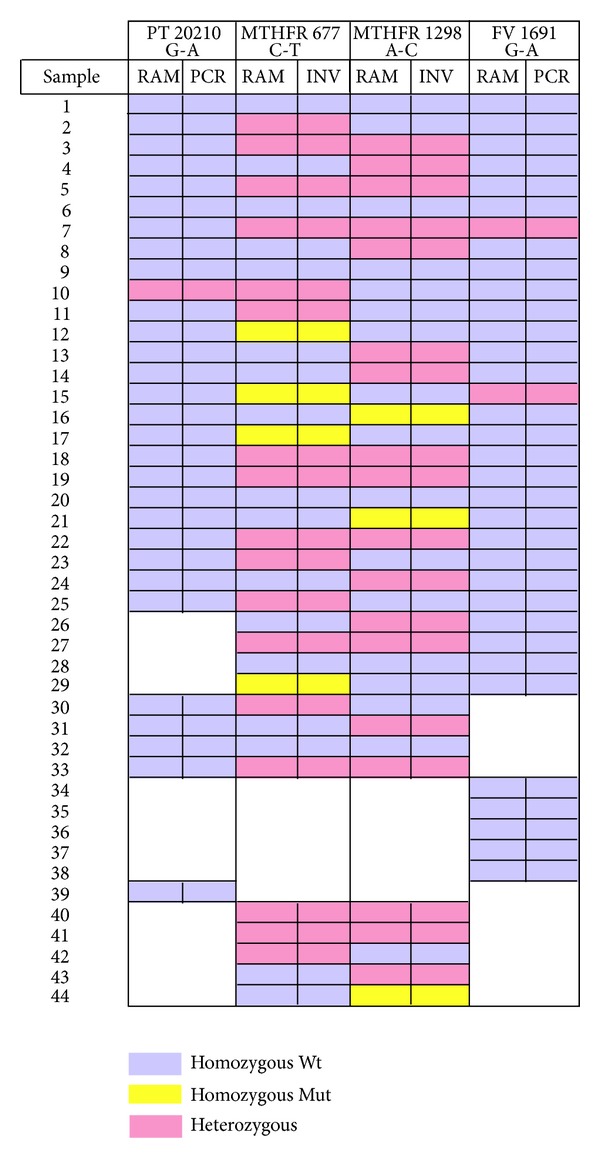
Comparison of genotypes scored after RAM reactions (“RAM”) versus genotypes called by commercial assays (“PCR” or Invader, “INV”). Results from each locus are shown in two columns; samples are shown in rows. Colors indicate each sample's genotype at each locus, as assessed by RAM assay (left columns) or FDA-cleared assay (right columns). Whitespace indicates that no test was performed on the indicated sample, assay pair.

A total of 280 allele determinations were made for comparison to results obtained from commercial assays. Although replicate RAM reactions were run as an internal consistency check, either of the RAM reaction series could have been used for comparison to obtain the same outcome.  Figure 5 shows graphically the results obtained after unblinding the commercial assay results. SNP allele determinations made after C-probe ligation and RAM reaction scoring were completely consistent with the commercial assay results over all reported alleles. The summary in Table 4 shows the results by genotype within each individual SNP category from the 44 patient samples tested. Of the samples tested for the MTHFR, more than half of the outcomes were either heterozygous or homozygous Mt. No homozygous mutants were detected for the FV and PT targets. The distribution of genotypes in Table 4 reflects the allele frequencies of the patient samples received in our laboratory where the MTHFR mutant alleles are more frequent than the FV and PT mutants (data not shown). In the clinical samples tested the Mt probes for FV and PT show good specificity; however, the low number of Mt alleles detected at the FV and PT loci in this study does not allow definitive evaluation of their clinical sensitivity but does demonstrate acceptable sensitivity with the control DNAs (Figure 3).

## 4. Discussion

Earlier we showed that target capture, C-probe hybridization, ligation, and RAM amplification can be configured on an automated platform, and the assay system could differentiate a single nucleotide difference in commercially available purified genomic DNAs. To further evaluate the RAM assay, we tested clinical samples of known genotype for 4 individual SNPs in 3 different genes. The 4 discrete SNPs are represented by 3 different nucleotide base substitutions; two SNPs are guanine to adenine (G>A) transitions; one is cytosine to thymidine transition (C>T) and the other is adenine to cytosine transversion (A>C). The format of the assay addresses each allele separately, employing 8 capture and C-probe pairs (Table 1) and utilizing independently both strands of the nucleic acid targets. The rationale for this approach was to achieve the most favorable mismatch, where possible, between the 3′ C-probe nucleotide and the SNP base. Favorable mismatches minimize the nonspecific ligation of C-probes on nonhomologous targets, which can result in one form of RAM assay noise (discussed below). However, the assay can be simplified in a format where both C-probes address the SNP allele on the same target strand (unpublished data).

The use of a capture probe and bead capture in the assay has several benefits. Clinical samples are generally processed to remove components that would inhibit enzyme activity and thereby impair assay performance [26]. Detergents, especially ionic types, used in sample processing [27, 28], can help liberate and protect targets released from cells. The binding and washing of the captured nucleic acid target complexes substantially reduce detergents, other components,and sample particulates prior to ligation and amplification. Use of a target specific capture probe in combination with a C-probe may also improve the specificity of the assay, particularly if there are closely related target sequences to which the C-probe may bind elsewhere in the genome. In addition, the use of a capture probe and magnetic beads reduces one other form of background noise (discussed below). These advantages were not fully utilized here in a demonstration of consistency with the existing sample processing.

There are several distinguishable nonspecific background noise sources in RAM assays [1]. Careful design and selection of C-probes and their corresponding primers significantly reduce primer interaction noise; in addition, using capture probes and beads in the assay significantly reduces unligated C-probe associated nonspecific RAM reaction product background noise [1].

The ligation of C-probes on nonhomologous SNP targets can also be a source of noise in RAM reactions. Those events are rare [14, 24] but can occur, resulting in ssDNA circles that are identical to circles formed after ligation on homologous template DNA. Likewise, RAM products generated from circles formed on nonhomologous targets, apart from their late Rts, are indistinguishable from homologous ligation signals and cannot be differentiated by product RAM product analysis from homologous target ligation signals. The assay as described seeks to minimize those reactions by optimizing the gene-specific ends of the C-probe, by the choice of target strand and C-probe (see below), and by limiting ligation reaction time and conditions.

Mismatch base pairings, and the context in which they exist, exhibit a range of thermodynamic favorabilities [29], and SNP discrimination should be greatest where the mismatch is least stable. For both G>A mutations (FV and PT), the plus strand (Wt SNP base = G) was chosen as the Wt target, that is, the WtC+, MtC− format, where the Wt C-probes are directed towards the Wt allele on the plus strand (Figure 2(a)). With this arrangement a mismatched C-probe and SNP base result in a C:A pairing. The alternative strategy, MtC+, WtC−, where the Mt C-probe hybridizes to the Mt allele plus strand, would result in a more stable G:T [29, 30] mismatch. In the case of the MTHFR SNPs, the MTHFR 677 C-T and the MTHFR 1298 A-C (C>T and A>C, resp.), the MtC+, WtC− design was employed to create C:A and A:G mismatches as opposed to G:T and C:T, respectively. In the MtC+, WtC− format the MTHFR 677 C-T base mismatch (C:A) is the same as would occur in PT 20210 G-A and FV 1691 G-A C-probe : target disparities. The format MtC+, WtC−, chosen for MTHFR 1298 A-C, resulted in a G:A mismatch. This difference was thermodynamically similar to the C:T mismatch that would be generated by the alternative WtC+, MtC− format [29]. We expect that minimizing ligation on a nonhomologous target should make ssDNA circle formation a rare event and should produce a response time that is much later than Rts from the equivalent homologous ligation. We did not compare both WtC+, MtC− and MtC+, WtC− probe: target formats for each SNP to test the prediction for base mismatch stability.

The lack of noise in nonhomologous assays makes postreaction signal analysis relatively straightforward; simple two-dimensional plots of Mt versus Wt response times allow ready visualization of homozygotes or heterozygotes (Figures 3 and 4).

Padlock probes, in the molecular inversion probe form, have been applied for SNP detection, initially by Hardenbol et al. [15] and are now widely applied. In these assays, thousands of SNPs are assayed from a single sample with an array readout. In the clinical lab, by contrast, the number of samples is usually much greater than the number of targets to be assayed, making the array approach uneconomical. The process described here is appropriate for a clinical laboratory with sufficient sample processing demands to warrant moderate scale automation. To our knowledge, the only comparable process description, where C-probes are annealed directly to genomic DNA and assayed by isothermal RAM reactions, is Faruqi et al. [14] although no automation solutions were presented there and other assay differences exist.

Each the C-probe ligation and RAM assay presented here may provide distinct advantages over other nucleic acid detection assay formats. C-probe annealing makes the recognition events that flank the SNP nonindependent, in contrast to annealing unlinked sequences as in PCR. Edwards et al. [31] demonstrated SNP determination in a hexaploid wheat where PCR SNP determination was not feasible. Although a long C-probe sequence is required, the probe concentration in each hybridization is low as once the 5′ terminus of the C-probe binds to the target, the local concentration of the 3′ terminus, in the proximity of the target, increases substantially. Magnetic bead processing on an automated platform makes the process as described here well-suited for moderate to high sample numbers, and further automation is possible. The combination of an automated platform with capture-probes that bind coated magnetic beads makes the assay workflow efficient, and the RAM reaction that detects ligated C-probe circles is low-noise, isothermal, and sensitive.

## 5. Conclusion

We have further evaluated a mostly automated, bead-based, isothermal RAM SNP assay by testing a panel of clinically relevant SNP targets in real clinical samples. In a blind study, we performed 280 SNP assays on 44 clinical samples and the results were in complete agreement with results from commercial FDA approved kits. These results suggest that this approach can meet a need in the clinical lab where moderate numbers of samples can be tested against specific clinically relevant panels of SNP targets.

## Figures and Tables

**Table 1:** Nucleotide sequences of C-probes, capture probes and primers.

Oligo Name	Sequence	Targeted locus	Targeted allele	Targeted strand	Oligo role
Cpr8FVWt+	GCCTGTCCAGGGATCTGCTCTTACAATACGAGAACACCCGATTGAG AGAGTTTGGAAGTGTAGGCGTGAAGTCCATAACACATACCTGTATTC CTC	FV 1691 G-A	Wt	+	C-probe
CPFV+	Bio-TCAGAATTTCTGAAAGGTTACTTC-Spacer C3	FV 1691 G-A	Wt	+	Target capture
Cpr8Fwd61_22	ACACTTCCAAACTCTCTCAATC	FV 1691 G-A	Wt	+	RAM Fwd Primer
Cpr8Rvs03_20	CTGTCCAGGGATCTGCTCTT	FV 1691 G-A	Wt	+	RAM Rev Primer

Cpr9FVMt−	AGGAATACAGGTATTTTGTCCTTGAAGTAACCCTCGTGAAAGCCCTA CTCTATGAAATCTTGTAGCAGGACTCCGTTTAGCAGCACTGGACAG GCA	FV 1691 G-A	Mt	−	C-probe
CPFV−	Bio-CCTCTGGGCTAATAGGACTACTTCTAATCTG-Spacer C3	FV 1691 G-A	Mt	−	Target capture
Cpr9Fwd73_21	GAGTCCTGCTACAAGATTTCA	FV 1691 G-A	Mt	−	RAM Fwd Primer
Cpr9Rvs87_21	TGGACAGGCAAGGAATACAGG	FV 1691 G-A	Mt	−	RAM Rev Primer

Cpr10PTWt+	GCTGAGAGTCACTTTTATTGGGAACCATAGAACTTCGTATCGCTCGC ACAAGAATCGTGTTTGAACAAGTAGGTGAATGGGCATTGAGGCTC	PT 20210 G-A	Wt	+	C-probe
CPPT+	Bio-CTGCCCATGAATAGCACTGG-Spacer C3	PT 20210 G-A	Wt	+	Target capture
Cpr10Fwd48_20	TGCGAGCGATACGAAGTTCT	PT 20210 G-A	Wt	+	RAM Fwd Primer
Cpr10Rvs4_24	GAGAGTCACTTTTATTGGGAACCA	PT 20210 G-A	Wt	+	RAM Rev Primer

Cpr12PTMt−	AGCCTCAATGCTCCCAGTGCTACGAGATAGACTGTGTCCTTCGGTG CGATGTTACCTTATGTGAATCACGATAAACTTCATTTGACTCTCAGC A	PT 20210 G-A	Mt	−	C-probe
CPPT−	Bio-CCGTGAAAGAATTATTTTTGTGTTTC-Spacer C3	PT 20210 G-A	Mt	−	Target capture
Cpr12Fwd57_20	AGGTAACATCGCACCGAAGG	PT 20210 G-A	Mt	−	RAM Fwd Primer
Cpr12Rev 5_20	TCAATGCTCCCAGTGCTACG	PT 20210 G-A	Mt	−	RAM Rev Primer

Cpr13MTH677Wt−	CGATTTCATCATCACGCAGCTTTTCTTTGGTTATTGAAAGGTTCATC GCTTAGTGATACCCCGTCGCCAGCACTCGTCATCGCCTACCTGCGG GAGC	MTHFR 677 C-T	Wt	−	C-probe
CPMTH677−	Bio-TTTGAGGCTGACCTGAAGCAC-Spacer C3	MTHFR 677 C-T	Wt	−	Target capture
Cpr13Fwd61_20	GGGTATCACTAAGCGATGAA	MTHFR 677 C-T	Wt	−	RAM Fwd Primer
Cpr13Rev16_20	GCAGCTTTTCTTTGGTTATT	MTHFR 677 C-T	Wt	−	RAM Rev Primer

Cpr14MTH677Mt+	CTCCCGCAGACACCTTCTCCTTCCACGACGGAGGTATTGACTCGGG ATTTCCCAAAAGCAACAGATGGTGCCCCTATGATAGATGATGAAATC GA	MTHFR 677 C-T	Mt	+	C-probe
CPMTH677+	Bio-GCGGAAGAATGTGTCAGCCTC-Spacer C3	MTHFR 677 C-T	Mt	+	Target capture
Cpr14Fwd 56_20	TTTGGGAAATCCCGAGTCAA	MTHFR 677 C-T	Mt	+	RAM Fwd Primer
Cpr14Rev 16_20	TCTCCTTCCACGACGGAGGT	MTHFR 677 C-T	Mt	+	RAM Rev Primer

Cpr15MTH1298Wt−	AAGTGTCTTTGAAGTCTTCGTTCTTTACCTCACCAAGTCATCGTCCG CTCGTAGATAATGATAAGTGAGTAAAGTTATGAATGTTCTACTCCAG TGAAGA	MTHFR 1298 A-C	Wt	−	C-probe
CPMTH1298−	Bio-GAGGAGCTGCTGAAGATGTGG-Spacer C3	MTHFR 1298 A-C	Wt	−	Target capture
Cpr15Fwd11_25	GAAGTCTTCGTTCTTTACCTCACCA	MTHFR 1298 A-C	Wt	−	RAM Fwd Primer
Cpr15Rev64_24	TTATCATTATCTACGAGCGGACGA	MTHFR 1298 A-C	Wt	−	RAM Rev Primer

Cpr16MTH1298Mt+	CTTCACTGGTCAGCTCCTCCCCTACAGCAAAGCGGCGTCTGTGCGA AGATAATGATAAGTGAGTAAAGTTATGGATGGACGAAGAAAGACACT TG	MTHFR 1298 A-C	Mt	+	C-probe
CPMTH1298+	Bio-ACCATTCCGGTTTGGTTCTCCC-Spacer C3	MTHFR 1298 A-C	Mt	+	Target capture
Cpr16Fwd 7_20	TGGTCAGCTCCTCCCCTACA	MTHFR 1298 A-C	Mt	+	RAM Fwd Primer
Cpr16Rev61_26	CACTTATCATTATCTTCGCACAGACG	MTHFR 1298 A-C	Mt	+	RAM Rev Primer

**Table 2:** C-probe and primer concentrations used in hybridizations and RAMs respectively. Primer naming conventions are given in [13].

Allele	C-Probe	C-Probe Concentration in 2.5X buffer (nM)	Primer pair	Primer concentration (uM) in elution buffer
FV 1691 G-A Wt	Cpr8FVWt+	0.4	Cpr8FVFwd61_22	2.5
			Cpr8Rvs03_20	2.2
FV 1691 G-A Mut	Cpr9FVMt−	0.25	Cpr9Fwd73_21	2.5
			Cpr9Rvs87_21	1.5
PT 20210 G-A Wt	Cpr10PTWt+	0.1	Cpr10Fwd48_20	1.7
			Cpr10Rvs04_24	1
PT 20210 G-A Mut	Cpr12PTMt−	0.125	Cpr12Fwd57_20	3
			Cpr12Rev05_20	1.8
MTHFR 677 C-T Wt	Cpr13MTH677Wt−	0.25	Cpr13Fwd61_20	3.6
			Cpr13Rev16_20	2.2
MTHFR 677 C-T Mut	CPMTH677Mt+	1.25	Cpr14Fwd56_20	2.5
			Cpr14Rev 16_20	1.5
MTHFR 1298 A-C Wt	Cpr15MTH1298Wt−	0.125	Cpr15Fwd11_25	1.2
			Cpr15Rev64_24	0.7
MTHFR 1298 A-C Mut	Cpr16MTH1298Mt+	0.25	Cpr16Fwd 7_20	1.4
			Cpr16Rev61_26	0.9

All capture probes concentrations in the 2.5X buffer were 2.5 nM.

**Table 3:** List of control DNAs and corresponding C-Probes used for SNP detection.

Coriell catalog	Coriell nucleic acid	C-probe	Locus	Allele	ThDx allele state
GM00536	NA00536	Cpr8	FV 1691 G-A	Wt	Homozygous, Wt
GM14899	NA14899	Cpr9	FV 1691 G-A	Mut	Homozygous, Mt
GM00536	NA00536	Cpr10	PT 20210 G-A	Wt	Homozygous, Wt
GM16000	NA16000	Cpr12	PT 20210 G-A	Mut	Homozygous, Mt
GM03469	NA03469	Cpr13	MTHFR 677 C-T	Wt	Homozygous, Wt
CD00024	CD00024	Cpr14	MTHFR 677 C-T	Mut	Homozygous, Mt
GM08369	NA08369	Cpr15	MTHFR 1298 A-C	Wt	Homozygous, Wt
GM00536	NA00536	Cpr16	MTHFR 1298 A-C	Mut	Homozygous, Mt
GM16028	NA16028	Cpr8	FV 1691 G-A	Wt	Heterozygous
GM16028	NA16028	Cpr9	FV 1691 G-A	Mut	Heterozygous
GM16028	NA16028	Cpr10	PT 20210 G-A	Wt	Heterozygous
GM16028	NA16028	Cpr12	PT 20210 G-A	Mut	Heterozygous
GM16028	NA16028	Cpr13	MTHFR 677 C-T	Wt	Heterozygous
GM16028	NA16028	Cpr14	MTHFR 677 C-T	Mut	Heterozygous
GM16000	NA16000	Cpr15	MTHFR 1298 A-C	Wt	Heterozygous
GM16000	NA16000	Cpr16	MTHFR 1298 A-C	Mut	Heterozygous

**Table 4:** Summary of assay results by genotype.

SNP target	Wt	Ht	Mt	Totals
FV 1691 G-A	32 (94%)	2 (6%)	0 (0%)	34
PT 200210 A-G	29 (97%)	1 (3%)	0 (0%)	30
MTHFR 677 C-T	17 (45%)	17 (45%)	4 (10%)	38
MTHFR 1298 A-C	17 (45%)	18 (47%)	3 (8%)	38

Totals				140
